# Addressing culture and context in humanitarian response: preparing desk reviews to inform mental health and psychosocial support

**DOI:** 10.1186/s13031-017-0123-z

**Published:** 2017-11-15

**Authors:** M. Claire Greene, Mark J. D. Jordans, Brandon A. Kohrt, Peter Ventevogel, Laurence J. Kirmayer, Ghayda Hassan, Anna Chiumento, Mark van Ommeren, Wietse A. Tol

**Affiliations:** 1 Department of Mental Health, Johns Hopkins Bloomberg School of Public Health, 624 North Broadway Rm. 888, Baltimore, MD 21205 USA; 2 Research & Development Department, War Child Holland, Amsterdam, the Netherlands; 3 Center for Global Mental Health, Institute of Psychiatry, Psychology and Neuroscience, King’s College London, London, UK; 4 Duke Global Health Institute, Duke University, Durham, NC USA; 5 Department of Psychiatry, George Washington University, Washington, DC, USA; 6 Public Health Section, United Nations High Commissioner for Refugees, Geneva, Switzerland; 7 Division of Social and Transcultural Psychiatry, McGill University, Montréal, QC Canada; 8 Culture and Mental Health Research Unit, Jewish General Hospital, Montréal, QC Canada; 9 Department of Psychology, Université du Québec à Montréal, Montréal, QC Canada; 10 Institute of Psychology, Health and Society, University of Liverpool, Liverpool, UK; 11 Department of Mental Health and Substance Abuse, World Health Organization, Geneva, Switzerland; 12 Peter C. Alderman Foundation, Kampala, Uganda

**Keywords:** Mental health, Psychosocial, Humanitarian, Emergency, Culture, Context, Desk review

## Abstract

Delivery of effective mental health and psychosocial support programs requires knowledge of existing health systems and socio-cultural context. To respond rapidly to humanitarian emergencies, international organizations often seek to design programs according to international guidelines and mobilize external human resources to manage and deliver programs. Familiarizing international humanitarian practitioners with local culture and contextualizing programs is essential to minimize risk of harm, maximize benefit, and optimize efficient use of resources. Timely literature reviews on traditional health practices, cultural beliefs and attitudes toward mental health and illness, local health care systems and previous experiences with humanitarian interventions can provide international practitioners with crucial background information to improve their capacity to work efficiently and with maximum benefit. In this paper, we draw on experience implementing desk review guidance from the World Health Organization (WHO) and UNHCR, the United Nations Refugee Agency (2012) in four diverse humanitarian crises (earthquakes in Haiti and Nepal; forced displacement among Syrians and Congolese). We discuss critical parameters for the design and implementation of desk reviews, and discuss current challenges and future directions to improve mental health care and psychosocial support in humanitarian emergencies.

Mental health and psychosocial interventions in humanitarian emergencies aim to promote and protect wellbeing, and prevent and treat mental disorders [1]. With growing consensus on best practices, mental health and psychosocial support is increasingly recognized as an essential feature of humanitarian response. However, a challenge for international humanitarian practitioners from different geographic, economic, and socio-cultural origins (i.e., “outsiders”) is ensuring timely service delivery that is appropriate to culture and context [2]. Ignoring information about existing services and socio-cultural context may result in inaccurate assessment methods, ineffective implementation of programs that the population does not want or use, and have potentially unintended consequences [3, 4].

Researchers have documented examples of cases where the provision of culturally inappropriate mental health care or psychosocial support in an emergency has had negative impacts on the target population. For example, in Albania, the provision of gender-based violence counseling to Kosovar survivors of war in the 1990s by a foreign psychologist resulted in public identification of sexual violence survivors, which was regarded as an insult to familial honor that could only be resolved by killing the survivor [5]. In another case, reintegration programs in Angola administered by traditional healers often focused on forgetting ones past; however, outsiders administered psychological debriefing, which encouraged survivors to speak about their trauma history, which may have undermined local approaches to recovery [5]. During a civil war in Nepal, posttraumatic stress disorder (PTSD) treatment programs went unused because the programs did not take language and cultural models of distress into account; the stigma associated with using the culturally oblivious programs was considered worse than the experience of posttraumatic distress [6]. During Sri Lanka’s protracted political violence, widows and other women who lost male relatives participated in mental health humanitarian programs were ostracized by community members because of the perception of violating norms to prevent future violence [7]. Also in Sri Lanka, after the 2004 tsunami many organizations arrived, often uninvited, to deliver psychosocial interventions. These activities generally lacked community consultation, coordination, qualified providers and an evidence-base, which confused and, in some cases, exacerbated distress among community members while also compromising the success of existing local programs [8–10]. Despite a general consensus supporting a culturally informed and sensitive response, existing cultural and contextual information is rarely utilized effectively to inform the design of programs delivered in humanitarian emergencies [11].

Academic literature to inform the development, selection and implementation of mental health and psychosocial interventions is generally not available in a format that is accessible and readily applicable by practitioners in humanitarian settings. There is broad agreement in the humanitarian community that secondary analyses of available data should be conducted before primary data collection to rapidly assess existing information, conserve limited resources, and avoid burdening emergency-affected populations by duplicating research [12, 13].

Desk reviews of existing literature are an efficient method to distil available knowledge to assist programmatic decision-making by (1) providing outsiders with a short synthesis of large bodies of literature, (2) delivering a high-quality document produced by experts trained in review methods, and (3) reducing the need for practitioners on the ground to collect background information that diverts time from service delivery priorities. Desk reviews can provide an important first step in achieving balance between internationally recommended interventions and appropriate local practices in situations where outsiders are assisting with mental health and psychosocial response.

## WHO-UNHCR desk review guidance

In 2012, the WHO and UNHCR published a toolkit with 15 tools for conducting mental health and psychosocial needs and resource assessments in humanitarian settings. The toolkit has been frequently used by a range of agencies, including non-governmental organizations in partnership with governmental or multilateral agencies, for example, to inform intervention development for refugees from South Sudan [14], to improve coordination among mental health and psychosocial providers in Jordan, Haiti, Nepal and Syria [15], and to identify data on mental health and psychosocial services for Syrian refugees in Lebanon [16].

This toolkit includes a guide for conducting desk reviews, often recommended as the first step in a needs assessment, which built on lessons learned from a review of mental health and culture written following the 2010 Haiti earthquakes [17, 18]. The guide provides a suggested outline or table of contents detailing the types of information that can be included in the review ranging from the socio-cultural context (e.g., demographics, economic data, formal and non-allopathic health systems, human resources) to idioms of distress, and existing services (Table 1). This breadth of information is included to provide insight into the types of clinical assessments, interventions and methods of implementation that may be feasible and acceptable in the setting. Desk reviews are intended to pragmatically synthesize academic and grey literature to deliver timely information in a readable style for practitioners (i.e., concise, pragmatic, avoiding jargon and theory), whose primary concern is service design and delivery. Literature searches may be complemented by interviews with experts to gather additional critical information.

**Table 1:** Sample Table of Contents for a Desk Review [18]

1. Introduction 1.1. Rationale for the desk review (description of current/recent emergency) 1.2. Description of methodology used to collect existing information (including any database search terms used)
2. General Context 2.1. Geographical aspects (e.g., climate, neighboring countries) 2.2. Demographic aspects (e.g., population size, age distribution, languages, education/literacy, religious groups, ethnic groups, migration patterns, groups especially at risk to suffer in humanitarian crises) 2.3. Historical aspects (e.g., early history, colonization, recent political history) 2.4. Political aspects (e.g., organization of state/government, distribution of power, contesting sub-groups or parties) 2.5. Religious aspects (e.g., religious groups, important religious beliefs and practices, relationships between different groups) 2.6. Economic aspects (e.g., Human Development Index, main livelihoods and sources of income, unemployment rate, poverty, resources) 2.7. Gender and family aspects (e.g., organization of family life, traditional gender roles) 2.8. Cultural aspects (traditions, taboo, rituals and practices related to health and well-being) 2.9. General health aspects 2.9.1. Mortality, threats to mortality, and common diseases 2.9.2. Overview of structure of formal, general health system
3. Mental health and psychosocial context 3.1. Mental health and psychosocial problems and resources 3.1.1. Epidemiological studies of mental disorders and risk/protective factors conducted in the country, suicide rates 3.1.2. Local expressions (idioms) for distress and folk diagnoses, local concepts of trauma and loss 3.1.3. Explanatory models for mental and psychosocial problems 3.1.4. Concepts of the self/person (e.g., relations between body, soul, spirit) 3.1.5. Major sources of distress (e.g., poverty, child abuse, infertility) 3.1.6. Role of the formal and informal educational sector in psychosocial support 3.1.7. Role of the formal social sector (e.g., social services) in psychosocial support 3.1.8. Role of the informal social sector (e.g., community protection systems, neighborhood systems, other community resources) in psychosocial support 3.1.9. Role of the non-allopathic health system (including traditional or indigenous health system) in mental health and psychosocial support 3.1.10. Help-seeking patterns (where people go for help and for what problems; who accompanies them; potential barriers to access) 3.2. The mental health system 3.2.1. Mental health policy and legislative framework and leadership 3.2.2. Description of the formal mental health services (primary, secondary and tertiary care). Consider the relevant Mental Health Atlas and WHO-AIMS reports among other sources to find out availability of mental health services, mental health human resources, how mental health services are used, how accessible mental health services are (for example distance, fee for service), and the quality of mental health services 3.2.3. Relative roles of government, private sector, NGOs, and traditional healers in providing mental health care
4. Humanitarian context 4.1. History of humanitarian emergencies in the country 4.2. Experiences with past humanitarian aid in general 4.3. Experiences with past humanitarian aid involving mental health and psychosocial support
5. Conclusion 5.1. Expected challenges and gaps in mental health and psychosocial support 5.2. Expected opportunities in mental health and psychosocial support
6. References

Since publication of the guidelines, several desk reviews have been conducted in response to humanitarian emergencies [19–26]. To assist future desk reviewers, this paper brings together the WHO/UNHCR toolkit developers and desk review authors to identify effective desk review procedures. We aim to illustrate the desk review process, and reflect on gaps and opportunities for refining current methods through four case examples from diverse geographic regions (Haiti, Nepal, Syria, Tanzania) and types of crises (earthquakes, armed conflict; Table 2). Based on our collective experience, we present considerations on critical parameters of desk reviews (i.e., for whom, when, where, what, why, who and how) and discuss how they may be tailored for different circumstances to rapidly inform humanitarian response.

**Table 2:** Summary of desk review methods used in four case examples

	When *Determining the timeline*	Where *Defining the target population and context*	What *Defining the question and scope*	Why *Defining the rationale and purpose*	Who *Selection of the research team*	How *Selection of data collection and synthesis methods*
Haiti (2010)	3 weeks	Persons living in Haiti affected by the earthquake	Haitian mental health and services	To inform immediate and long-term efforts to improve mental health services in post-earthquake Haiti	19 experts and student volunteers	Rapid review of academic databases to retrieve peer-reviewed and non-academic literature; Hand search of key resources
Nepal (2015)	8 weeks	Persons living in Nepal affected by the earthquake	Mental health and psychosocial wellbeing in Nepal	To serve as a resource for humanitarian actors responding to the 2015 earthquakes in Nepal	98 experts and volunteers	Rapid review of academic literature, agency websites and expert recommended resources; WHO/UNHCR MHPSS Toolkit Desk Review methods and structure; “Crowd writing”
Syria (2015)	96 weeks	Persons residing within Syria and Syrian refugees affected by armed conflict	Cultural aspects of mental health and psychosocial wellbeing	To serve as a resource for MHPSS staff working with conflict-affected Syrians to assist in design and delivery of interventions	68 experts	Review of academic and non-academic literature and reports; Hand search of key resources
Tanzania (2016)	52 weeks	Adult female refugees from Eastern DRC with a history of SGBV affected by armed conflict	Mental health and psychosocial wellbeing in the context of SGBV in Congolese populations	To inform the development of a mental health and psychosocial intervention and the protocol for a randomized trial for Congolese survivors of SGBV in Nyarugusu refugee camp, Tanzania	5 researchers/study investigators and staff	Review of academic literature and agency websites; Iterative drafting and revision process among investigators

Abbreviations: DRC, Democratic Republic of the Congo; IASC, Inter-Agency Standing Committee; MHPSS, mental health and psychosocial support; SGBV, sexual and gender-based violence; UNHCR, United Nations High Commissioner for Refugees; WHO, World Heath Organization

Access to desk reviews: Haiti (http://www.who.int/mental_health/emergencies/culture_mental_health_haiti_eng.pdf); Nepal (https://interagencystandingcommittee.org/system/files/20150622_nepal_earthquakes_mhpss_desk_review_150619.pdf); Syria: (http://www.unhcr.org/55f6b90f9.pdf); Tanzania (http://mhpss.net/?get=294/mental-health-and-psychosocial-wellbeing-in-congolese-refugee-survivors-of-gender-based-violence.pdf

## Conducting desk reviews (Table 3)

**For whom:** The purpose of a desk review is to present information that can be used by humanitarian practitioners. This overall aim should guide what information is emphasized and how it is presented. Information that can be directly translated into mental health or psychosocial programming (e.g., local non-stigmatizing terms for mental health, cultural coping strategies) or that can inform practical decisions in the field (e.g., identifying vulnerable groups) should be prioritized and succinctly presented in a way that can be easily understood by practitioners. This is in contrast to the aim of conventional academic systematic reviews, for which the objective is usually to comprehensively address a well-defined research question and rigorously examine the methodological features of included research [27]. While also intended to inform practice, the translation of results from academic reviews to practice usually happens through later production of guidelines or recommendations aimed at practitioners. In contrast, the desk reviews described in this paper are intended to serve as an information source that can be immediately useful to practitioners. For example, the Nepal desk review was distributed directly to practitioners working in post-earthquake Nepal via the humanitarian cluster system. This important difference informs the structure, content and language used in such desk reviews. Ensuring the review is accessible to practitioners also requires dissemination of the desk review in both English and local languages, as was done in the Haiti and Syria reviews due to the large number of local mental health and psychosocial providers who did not read English.

**Table 3:** Considerations for developing and conducting desk reviews

For whom: Dissemination	
1.1	What is your plan for dissemination? Who is the primary end-user of the desk review and how will you ensure that they receive it?
1.2	What language is most commonly spoken by the end-users? Will the desk review need to be translated? Who will do the translation and who will check it?
1.3	What is your plan for evaluating the application and utility of the desk review in informing humanitarian response?
When: Determining the timeline	
2.1	When does this desk review need to be available to be useful for reasonably informing humanitarian response?
Where: Defining the target population and context	
3.1	Where is the target population and how are they defined in terms of geographic, social, and cultural characteristics?
3.2	Which sociocultural factors require particular attention?
Why: Defining the rationale and purpose	
4.1	Why has the particular agency or stakeholder requested the review at this time?
4.2	What is the purpose/objective(s)? How will it be used?
4.3	What gaps in knowledge among humanitarian responders will this desk review fill?
What: Defining the question and scope	
5.1	What aspects of mental health and psychosocial wellbeing, socio-cultural context and humanitarian response are most relevant to the context and purpose of the desk review?
5.2	Information from which populations (person, place and time) and issues might provide relevant data to inform mental health and psychosocial practices in the target population?
Who: Selection of the research team	
6.1	How many people are needed to achieve the objectives/purpose within the timeline?
6.2	Are there experts on mental health and psychosocial support from the specified target population/context available and able to contribute?
6.3	If the conduct of the desk review requires a large team(s), how will communication and tasks be coordinated?
6.4	What regional experts and stakeholders will review the report?
How: Selection of data collection and synthesis methods	
7.1	What data sources (e.g., academic, websites, agency reports, news sources, etc.) are relevant to the scope of the desk review?
7.2	What languages need to be included in the search?
7.3	What (combination of) search terms will identify the relevant literature?
7.4	Does the search strategy produce an adequate and manageable number of results (maximize sensitivity and specificity) relative to the resources available to conduct the review?
7.4	How will search results be documented?
7.5	How will literature be literature be reviewed to extract and synthesize relevant information? (e.g. use of structured recording forms)
7.6	How will reading and writing tasks be organized among team members. How will the final report be edited and formatted?
7.7	What level of detail is important for each section of the desk review?
7.8	Who will edit the final report for accuracy and consistent style?

**When:** Desk reviews must be completed in a timeframe that meets the needs of potential users, which may vary depending on whether the humanitarian response around mental health and psychosocial support commences in the acute response period or whether activities commence in a protracted emergency situation. The decision to conduct a desk review in the examples listed here was precipitated by the onset of an emergency (e.g., earthquakes in Haiti, Nepal; armed conflict in Syria) or a mental health initiative in a protracted refugee setting (e.g., Congolese refugees in Tanzania). Determining the timeline requires consideration of the purpose, scope and available resources (e.g., personnel, time). In the examples, the timeline varied from 3 weeks (Haiti) to 96 weeks (Syria). To provide a rapid response to the need for information after the earthquake in Haiti, the lead author/editor (LJK) assembled a group of clinicians and scholars familiar with literature on culture and mental health in Haiti. Assembling a large team of volunteers for the Nepal review greatly assisted in completing a comprehensive desk review draft for peer-review in 4 weeks, which was disseminated 4 weeks later. A short timeline is essential in sudden onset crises so that dissemination occurs within the period where critical humanitarian decision-making is taking place and before the situation drastically changes. In contrast, the Congolese refugee desk review was conducted for a population that had resided in a Tanzanian refugee camp for approximately 20 years. Here there was arguably less urgency, as humanitarian activities were not likely to fundamentally change in the period of several months. The longer timeline of the Syria review reflected the decision to prioritize detailed examination of the review by a variety of experts and institutions.

**Where:** Desk reviews should include clear guidance about the extent to which statements can be generalized to the affected populations given information available and changing circumstances. For example, the Nepal desk review noted that applying the summarized knowledge should be done with caution given the country’s socio-cultural diversity and that most available literature focused on populations affected by political violence rather than earthquakes. Other reviews may be restricted to a specific target population, such as people proximal to the location of a disaster or heavily conflict-affected area or populations that lacked access to health care even before the disaster. The Congolese refugee desk review, for example, focused specifically on female survivors of gender-based violence. The purpose of this desk review was to inform the development of mental health and psychosocial programming specifically for women with a history of gender-based violence, whose experiences and psychosocial needs may differ from those of the general Congolese refugee population. Thus, the results of this desk review were not intended to be generalizable beyond this population.

**What & Why:** The scope of desk reviews is best defined by the need for specific knowledge to inform program design and service delivery. Humanitarian practitioners need a short practical document covering a range of issues. A recent review on relevant public health information relevant in crisis situations recommended including information about the affected population size, age, gender structure and composition [11]. Contextual features such as economic and social structures, transportation infrastructure, communication resources and other factors may assist in anticipating and circumventing implementation challenges [28]. Cultural factors such as idioms of distress and help-seeking patterns may provide insight into the acceptability and utilization of mental health and psychosocial interventions [6]. If time allows, review teams should consult with practitioners in the design phase to determine which types of information would be most helpful, recognizing that a focused summary of key cultural factors influencing health is more relevant than lengthy descriptions of cultural practices and beliefs. To facilitate uptake, reviews should include an executive summary and key points for mental health and psychosocial support practitioners.

**Who:** The scope of the review and size of the literature will dictate which data collection and synthesis methods are feasible, as well as the required size of the desk review team. Desk reviews do require considerable time and effort, and producing results quickly may require assembling a large group, which may include volunteers with relevant background knowledge who donate their time as a way to help with the crisis. Although some aspects of the review (e.g., database searches and article summaries) can be carried out by students or research assistants familiar with general issues in culture and mental health, it is essential to include persons knowledgeable about mental health and psychosocial support in the specific population affected by the humanitarian crisis. These experts can assist in setting the scope of the desk review, identify gaps, share relevant information and resources, validate the content, highlight key practice issues, and provide additional context where necessary. For example, in the Syria review, the contribution of Syrian authors from various religious and ethnic backgrounds was crucial for describing idioms of distress in the Syrian dialect of Arabic and the Kirmanji Kurdish language, and in reducing over-generalizations and stereotyping. The Nepal review included consultation with Nepali mental health professionals living in the United States who had time to provide constructive input whereas Nepali mental health professionals in Nepal were engaged in full time relief activities. Involving native speakers enhances reviewing and interpreting literature published in the local language(s). The inclusion of glossaries – as was done for the Nepal review – with common local mental health terms and recommended local translation of mental health and psychosocial support terminology (e.g., trauma, flashbacks, resilience) can reduce the risk of stigmatizing or misleading translations).

Triangulating the information included in a desk review through input from a range of mental health and local experts is essential to verifying its accuracy and presenting the content in a manner that avoids propagating myths, assumptions and harmful stereotypes. Moreover, including humanitarian practitioners will help strike a balance between providing too much detail and potentially harmful over-generalizations.

**How:** Prior to data collection it is important to identify sources of information that will provide the breadth and depth necessary for the specific goals of the review. Data and reports from agencies (e.g., governmental, non-governmental, humanitarian) can be useful in providing up-to-date information on the humanitarian situation and existing programs. For example, the Inter-agency Information Sharing Portal for the Syria Regional Response and the website of the Assessment Capacities Project (ACAPS), a humanitarian organization specialized in secondary data analyses, are rich sources of timely data on humanitarian emergencies and responses [29, 30]. Data from social media and other communication platforms may also be used to gather information in real-time on rapidly evolving contextual factors. Peer-reviewed literature, including publications in the language(s) spoken by the target population, is helpful for providing historical context, the evidence-base for mental health and psychosocial interventions, and prior experiences with humanitarian response. Ethnographic monographs, book chapters and theses can also provide useful information, but require more time to synthesize. Engaging scholars familiar with this literature can facilitate efficient inclusion of such material. For example, leaders of the Nepal review asked medical anthropologists to summarize long-standing ethnographic research experience in the form of key points that were integrated into the review. One Nepali scholar specializing in Tibetan medicine provided details related to management of psychological distress and the psychological impact of destruction of religious sites, which had occurred throughout earthquake-affected areas.

Search strategies should be tailored to the overall objective to provide a targeted review that covers essential sources. Guidance on the development of search strategies for systematic reviews is publicly available online and can be customized to fit the scope of a desk review [27]. In the Nepal review, which had a broad scope, inclusive search strategies were developed. For example, search strategies for grey literature databases were as simple as “Nepal” or “Nepal mental health psychosocial”. Searches of academic databases were also simple, but utilized Boolean operators to increase the relevance of the returned records. The Haiti review included searches on Google Scholar, which facilitated access to book chapters, theses and other resources not always indexed in academic databases, supplemented with bibliographies from previous work. The Syria review included focused searches for information on culture-specific idioms of distress identified through an initial scoping literature review and consultations with experts.

For desk reviews summarizing a large body of literature, search terms may need to be refined to ensure that the search yields a representative, but manageable number of records. In rapid desk reviews (e.g., Haiti, Nepal), database searching and screening tasks were distributed across team members. In desk reviews with a generous timeline or limited scope (e.g., Congolese refugee review), it was possible for one or two team members to complete the searches and screening.

Similarly, data management and synthesis procedures may be tailored to the scale of literature and size of the team. In the Nepal review, which employed a large team across different institutions, data management procedures were critical. Data management and synthesis was conducted using shared documents on an online cloud drive. The cloud drive had a folder system that was organized according to the WHO/UNHCR toolkits’ desk review guide for a suggested table of contents (Table 1). The institutions and team members conducting the Nepal desk review were recruited to participate by the three investigators (WAT, BAK, MJDJ) who initiated and led the review. Each institution had a coordinator, who directed the team members affiliated with that institution. The volunteers searched different databases and noted results in shared spreadsheets. Each eligible document was indexed to relevant section(s) of the table of contents. Coordinators then assigned volunteers to summarize articles that were indexed to a given topic. These summaries were subsequently edited for clarity and consistency by the investigators, who divided topics according to their expertise. In this scenario, it was essential to develop detailed instructions for volunteers to maintain consistency in procedures (available upon request from authors). This method, referred to as “crowd-writing”, allowed multiple volunteers to collaborate in real-time. The benefits of this procedure must be weighed against the substantial challenge of managing 98 volunteers working simultaneously on living documents, which requires structured planning and communication. A text editor with in-depth anthropological knowledge of the context was needed to develop consistent formatting, grammar, terminology and translations, and writing style. The three investigators and others invited for peer-review then examined the document in its entirety.

Another strategy is to complete sections of the desk review through an iterative process of revisions incorporating the input of multiple contributors in sequential drafts, as was the case for the Congolese refugee and Syria reviews. Advantages of this strategy include that it allows for a single author to develop a cohesive document from the start instead of relying on an editor to provide consistent style at the end, and that the writing process can reflect consensus brokered among authors from various professional, ethno-political and religious backgrounds.

## Dissemination of desk reviews

Dissemination of the reviews occurred primarily via national and international humanitarian response and mental health and psychosocial support coordination groups, social media, and the social networks of desk review teams. More recently, a centralized, publicly accessible repository for mental health and psychosocial support desk reviews has been created at www.mhpss.net, which currently contains ten reviews [31]. Systematic tracking of accessibility and utilization has not been conducted to date, but is an important area for future research on knowledge translation to evaluate the applications of desk reviews in humanitarian practice. As emerging technologies propel humanitarian response towards increased connectedness [32], tracking the dissemination, uptake and utilization of desk reviews may become more feasible. Furthermore, social media platforms may be used strategically to increase awareness of desk reviews and expand their reach to practitioners operating outside of mainstream humanitarian systems.

## Challenges and future opportunities

The variation in procedures across desk reviews reflects decisions made by lead investigators about the critical parameters presented in this paper to address different humanitarian scenarios. Future desk review investigators will need to reassess these parameters. For example, a recent desk review in response to the West Africa Ebola epidemic focused on mental health and psychosocial considerations, but expanded the scope to include Ebola-specific concerns [26]. Desk reviews can also be useful beyond traditional humanitarian settings, such as in working with refugee populations resettled in high-income countries or with other diverse populations. These applications may require further adaptation of the procedures presented in this paper [33].

A central challenge for all of the desk reviews concerned the need to go beyond vague generalizations (e.g., “family is important to refugees in …”) to discuss specific cultural issues of critical relevance for programming (e.g., discussing mental health problems in terms of the brain-mind [*dimaag*] is potentially highly stigmatizing to Nepali speaking earthquake-affected persons). At the same time, however, presenting specific examples may inadvertently lead to stereotyping. There is a risk that desk reviews over-generalize from cultural examples to produce rigid, static frameworks of local experience (e.g., “The Syrian concept of depression is…”) that ignore variation in the population. There is heterogeneity and ongoing shifts in values, beliefs, knowledge and practices in any community [34]. This is especially true in areas with great linguistic diversity, such as Nepal where the earthquake-affected districts included native speakers of more than 25 languages [35].

Another example concerns idioms of distress (i.e., emotional distress expressed through locally salient metaphors and expressions) [34, 36–38]. In describing such idioms, it is important to be both concise and accurate, and where possible, specify the situations and subcultural groups where these idioms are used. Specific examples presented in the review should be framed as illustrations that suggest lines of inquiry in clinical assessment or intervention. The DSM-5 Cultural Formulation Interview presents a framework for contextualizing illness experience that can be used to describe cultural information and relate it to mental health assessment and intervention [39]. The involvement in the desk review of experienced social scientists and local experts with an intimate knowledge of the cultural context is essential to address these challenges.

Other important questions regarding desk review procedures and dissemination include how to improve existing methods to address current limitations such as timeliness, the potential for bias or stereotyping, accessibility, and challenges associated with translating the content into practice. Desk reviews need to be made rapidly available to practitioners and periodically reviewed and updated. While the WHO/UNHCR’s toolkit [18] desk review guidelines recommend that they be completed within 7 to 10 days from the onset of an emergency, none of the desk reviews produced to date were able to adhere to this timeline. Innovative procedures to improve efficiency are needed. In preparation for potential emergencies and to address existing gaps that have been prioritized by humanitarian organizations [40], relevant desk reviews may be commissioned. Internet-based templates and tools can be developed and made available for future use by desk review teams. Desk reviews conducted beyond the acute phase of an emergency (e.g., Syria and Congolese refugee desk reviews) are able to include information on how the ongoing crisis and response has changed the views and practices regarding mental health and psychosocial wellbeing in the population. Another strategy is for desk reviews to function as living documents, with built-in methods for ongoing editing and public contributions, like wiki-based websites, making it possible for reports to be delivered more quickly in partly finished format. A potential advantage of this approach is a reduction in the bias that may be introduced by a smaller review team. A major drawback is the risk of inaccuracies or inconsistencies and need for a qualified moderator to edit and manage content.

Questions have been raised about whether desk reviews should include recommendations for practice. When WHO commissions desk reviews (e.g., Haiti), it specifies that they should not include any recommendations. Unlike formal guidelines, desk reviews are based neither on wide consensus, nor on structured evidence synthesis methods [41]. In an effort to provide culturally-specific recommendations, desk reviewers may emphasize specific interventions, which may contrast with international guidelines (e.g., IASC guidelines [1]). In some cases, reviewers may endorse interventions that lack an adequate evidence-base or are contentious in other ways. Moreover, it can be confusing to have multiple sets of recommendations in one emergency setting, which may occur if the desk review is disseminated by agencies that have also endorsed other guidelines. On the other hand, inclusion of recommendations can make the implications of the desk review clearer and contribute to more culturally informed practice. Including a range of good practices, and indicating gaps in knowledge or possible omissions will reduce the risk of bias. Linking desk reviews to consensus-based guidelines and reviews of evidence-based practices (e.g., Evidence Aid reviews [42]) may improve uptake and appropriate application, without making specific recommendations.

Although this paper reflects the extensive experience of the authors, the recommendations are limited by the lack of information about the uptake and impact of desk reviews. While prior desk reviews have been distributed systematically to humanitarian actors, methods of desk review dissemination have, thus far, precluded our ability to track utilization, usefulness and effects on local services and outcomes. Furthermore, research has not been conducted to actively inquire about the uptake, utility and impact of these reviews to humanitarian practice. Evaluation research on the use and impact of desk reviews can help to refine effective methods, formats and dissemination strategies. We recommend that authors of future desk reviews engage with practitioners to determine whether and how the desk reviews are utilized, identify their strengths and limitations, and clarify how they influence practice.

## Conclusion

Desk reviews provide a pragmatic starting point for humanitarian mental health and psychosocial response by making existing information on culture and context available to international practitioners [43]. The strengths of desk reviews include the integration of multiple sources of knowledge and timely delivery of program-relevant information. The limitations of desk reviews include their dependence on existing literature, which may be susceptible to publication and reporting biases and the possibility that time–pressure precludes comprehensive coverage [44]. Additionally, it is important that desk reviews not be interpreted as providing the same strength of evidence as systematic reviews that have undergone peer-review. Desk reviews provide important information but are, by themselves, insufficient to develop culturally relevant and effective programs. They must be complemented by primary data collection and local stakeholder involvement to inform program design, as described in the WHO/UNHCR toolkit [18]. We anticipate that desk review procedures will continue to evolve through the experiences of other teams to address some of the current limitations, thereby improving our ability to provide timely information to mental health and psychosocial practitioners and by bridging the divide between research and practice in humanitarian emergencies.

## Abbreviations

DRC: Democratic Republic of the Congo
DSM-5: Diagnostic and Statistical Manual of Mental Disorders, 5th Edition
IASC: Inter-Agency Standing Committee
MHPSS: Mental health and psychosocial support
SGBV: Sexual- and gender-based violence
UNHCR: United Nations High Commissioner for Refugees
WHO: World Health Organization
